# The Use of Refuges by Communally Housed Cats

**DOI:** 10.3390/ani5020245

**Published:** 2015-04-24

**Authors:** Adriana Sicuto de Oliveira, César Augusto Sangaletti Terçariol, Gelson Genaro

**Affiliations:** 1 Programa de Pós-Graduação em Psicobiologia, Departamento de Psicologia, Faculdade de Filosofia, Ciências e Letras de Ribeirão Preto, Universidade de São Paulo, Av. dos Bandeirantes, 3900, Ribeirão Preto, SP 14040-901, Brazil; E-Mail: gelsongenaro@hotmail.com; 2 Centro Universitário Barão de Mauá, R. Ramos de Azevedo, 423, Jardim Paulista, Ribeirão Preto, SP 14090-180, Brazil; E-Mail: cesartercariol@gmail.com

**Keywords:** animal welfare, use of space, environmental enrichment, refuges, cats

## Abstract

**Simple Summary:**

Captive domestic cats frequently suffer from the lack of physical space and opportunities to perform species-typical behaviors, such as climbing or hiding. Environmental enrichment is a technique that helps transform the space available to animals into a more appropriate habitat. In this study, we tested horizontal and vertical refuge boxes as environmental enrichment for cats living communally in a cat rescue shelter. The provision of boxes in the environment increases the use of available space by the cats. We suggest this improves the cats’ welfare while in communally-housed rescue shelters.

**Abstract:**

The increase of domestic animals kept in shelters highlights the need to ensure animal welfare. Environmental enrichment can improve animal welfare in many ways, such as encouraging captive animals to use all the space available to them. The effects of physical environmental enrichment on the spatial distribution and behavioral repertoire of 35 neutered domestic cats housed communally were analyzed. The provision of boxes in the environment increases the use of available space by the cats. We suggest this improves the cats’ welfare while in communally-housed rescue shelters. The frequencies of active and especially inactive behaviors also increased in the enriched condition. In a test with vertical environmental enrichment, the animals showed an increased length of stay in refuges located at a height of 0.5 m compared to those on the ground (0.0 m). However, the entry frequency was higher in refuges at 0.0 m. Both horizontal and vertical environmental enrichment increased the use of available space, demonstrating that box refuges as enrichment are effective in providing a refuge when at a height, or a place to explore at ground level. We suggest it enhances the welfare of cats in communally housed shelters. This information adds to the body of evidence relating to cat enrichment and can be useful in designing cat housing in veterinary clinics, research laboratories, shelters and domestic homes.

## 1. Introduction

The number of confined domestic cats has increased and includes those in homes, shelters [1,2], veterinary clinics and animal facilities of research institutions. Domestic cats adapt well to confinement [3,4]; however, animals kept in confinement for a long period of time need a sufficiently complex environment that allows them to meet most of their needs, such as exercise and social interaction [5].

A captive environment imposes spatial restrictions that affect the behavioral repertoire of animals [6] and possibly their welfare [7]. It may influence, for example, the levels of agonistic interactions [1,8] and stress and consequently reduce the quality of life [9]. Cats in poor welfare conditions often urinate/defecate in undesirable locations [1], which is one of the main complaints of their owners [10]. Large enclosures allow animals to choose social groups, avoid aggressive individuals and increase their chances of escaping from these individuals [11]; being offered sufficient space makes it possible keep distances of between 1 and 3 m from one cat to another [8].However, the excess of homeless cats leads some shelters to accept more animals than possible [12], resulting in overcrowding [13]. Nonetheless, the quality of the space that houses an animal depends on more than its size [4,14]. Animals in simple captive settings live in highly predictable environments and have few chances to successfully cope with daily challenges [15].Environmental enrichment techniques are introduced to improve welfare through providing environmental changes [16], either in structure or content [17,18].

Environmental enrichment increases environmental complexity [19] and can enhance welfare by simply offering new opportunities for animals to express behaviors that were previously suppressed [18], thereby reducing abnormal behaviors [18,20,21,22].Enriching an environment, including modifying the use of an available physical space that cannot be structurally expanded, stimulates exploratory behavior in captivity. Mice (*Mus domesticus*), for example, tended to use space less efficiently when the space lacked structural complexity [23], and chickens (*Gallus gallus domesticus*) began to use central areas that had previously been avoided after the implementation of vertical panels on farms [24]. Cats started approaching humans when they had more hiding places available, which may indicate a decrease in anxiety levels [25]. Indeed, studies on how animals use an environment are valuable for assessing its appropriateness [6].

The use of boxes as enrichment provides the opportunity for the cat to hide, a behavior that is considered a necessity for cats exposed to stressors [26] and is even part of their biology [27]. Many studies have been conducted with the implementation of shelters for solitary cats kept in shelters or laboratories, resulting in a decrease in cortisol levels [28] or even lower stress scores (Cat-Stress-Score, [29]) [25,27]. Boxes with the possibilities of internal and superficial exploration may also increase the available area for resting where it is not possible to increase the floor area [30]. However, there are still no studies evaluating changes in the use of available space from environmental enrichment with boxes. Similarly, although Vinke *et al.* [27] and Kry and Casey [25]emphasize the importance of such studies with cats kept in groups, so far we only have results with cats tested alone [13,25,27,28,30].

The possibility of the vertical exploration of an environment through the implementation of elevated structures also relates to the welfare of domestic cats [8,26]. With the opportunity to explore different levels, cats are more likely to climb and jump [26]. Different heights also allow cats some control over the environment because they gain a larger field of vision [28]. However, most of the studies related to the use of vertical space by domestic cats only deal with platforms and not refuges, thus not considering the importance of the behavior of hiding within the vertical space.

The aim of this study was to evaluate the effects of environmental enrichment with boxes in the use of the space previously available for cats as well as the use of vertically provided refuges. We hypothesized that there would be an increase in the use of enriched space and preference for higher refuges by animals.

## 2. Materials and Methods

The study was approved by the Animal Ethics Committee of the University of São Paulo (protocol No. 10.1.651.53.4, 5 August 2010) and was based on another study that was completed at the same location [31]. Thirty-five neutered cats (12 males and 23 females) at an animal welfare shelter were used. The animals had lived in the same enclosure for at least three years, and their precise ages were unknown. All the animals were vaccinated regularly, and none died or showed signs of diseases during the study. Dry adult cat food and fresh water were available *ad libitum*.

**Figure 1:**
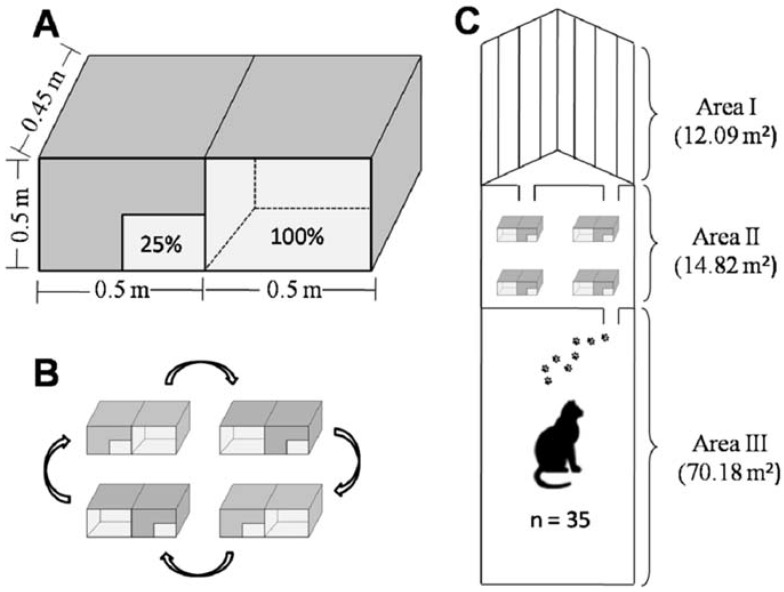
(**A**) Representation of pairs of cardboard refuges used in Experiment I; (**B**) Diagram with arrows that indicate the rotation applied to each day of Experiment I; (**C**) Sketch of total area available for cats observed in this study.

The enclosure under study was divided into three areas: an internal area (Area I: 12.09 m^2^) that housed the beds and feeders and was protected with a masonry roof, a second smaller external area (Area II: 14.82 m^2^) that was surrounded by fencing and was the site of video recording and a third external area (Area III: 70.18 m^2^) (Figure 1). The animals could freely access all three areas.

The internal area I was linked to area II by doors, and area II could be isolated from Area III by closing a gate. Area II was isolated when it was necessary to use the feeding area for administration of medication or to set up experiments; this area did not have any environmental enrichment. Area III had tables, shelves, wood and sisal scratching posts as permanent enrichments. The animals were tested in groups as the animals in the study lived in relatively stable groups and ultimately the enrichment would be available when the cats were in groups, so it was important to observe their behavior in experimental conditions similar to an applied setting.

### 2.1. Experimental Protocol

#### 2.1.1. Experiment I: Use of Horizontal Space

A camera was installed in Area II that recorded the use of the space without environmental enrichment (“control environment”) for four days between 15:30 h and 17:30 h. The animals were kept outside the feeding area two hours before the beginning of the test (13:30 h). This procedure was adopted to allow the setup of boxes, refuges and other components of the experiment in the other conditions. At 15:30 h, the gate dividing Areas II and III was opened, allowing the animals free access to the area where the video recording took place.

In the experimental condition (“enriched environment”), while the animals remained confined in Area III (from 13:30 h to 15:30 h), four pairs of cardboard refuges (boxes) (50 cm × 45 cm × 45 cm) were placed in Area II, with each pair being composed of one refuge with a 100% lateral opening and another with a lateral opening of only 25%. Each test day, the pairs were rotated because witching the position of the boxes with 100% and 25% openings would reduce the chances of possible bias(Figure 1). The video recording started at 15:30 h, and the gate dividing Areas II and III was opened as in the control situation, thereby allowing all the animals to enter the video recording area; thus, all individuals had the same opportunity to interact with the refuges. The area enriched with the refuges was also recorded for four days between 15:30 h and 17:30 h, and when the video recording ended, all the boxes were removed from the enclosure. In both stages, enriched and non-enriched, the video recording was monitored from a distance, and no one entered the enclosure during the test.

#### 2.1.2. Experiment II: Enrichment of the Vertical Space

**Figure 2:**
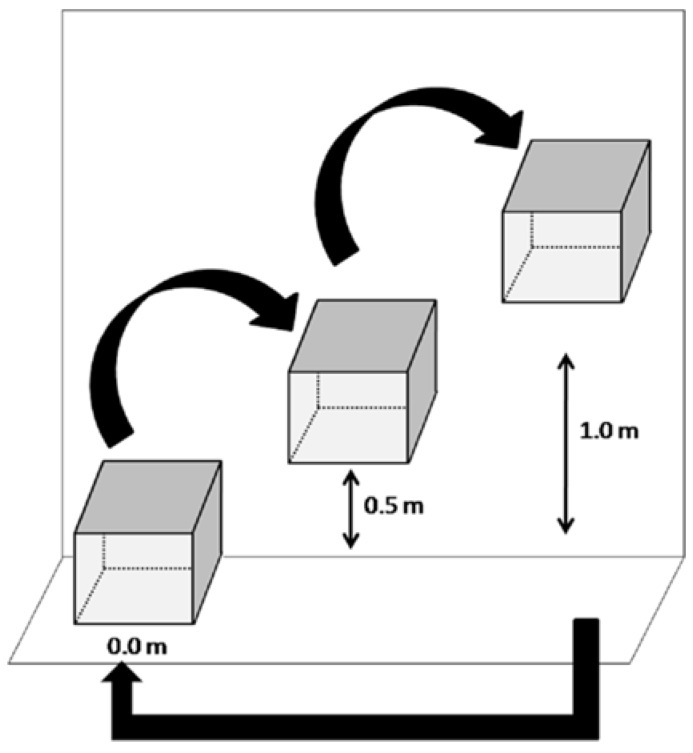
Diagram of the experimental set up of Experiment II. The arrows represent rotation of the boxes in three different positions at the wall, applied to each day of testing.

The same refuges as in Experiment I were used for this enrichment, and the animals were again kept outside of the feeding area for two hours before the beginning of the test (13:30 h), allowing for the setup of the refuges in the test environment. The refuges were attached with screws to the walls at various heights (0.0 m, 0.5 m and 1.0 m). During the three days of testing, the experimental protocol was the same: at 15:30 h, the gate allowing access to the feeding area was opened, and images were recorded for two consecutive hours. However, a height rotation was performed every day so that the three heights (0.0 m, 0.5 m and 1.0 m) were tested at three different locations—A, B and C(Figure 2). The frequency and the duration of both the use of the refuges and other types of interactions with them were quantified.

The boxes and the shelves were removed from the environment at the end of the two-hour period of recording to minimize possible effects of habituation.

### 2.2. Data and Statistical Analysis

#### 2.2.1. Experiment I: Use of Horizontal Space

During the analysis of the images, the number of animals in the area every five minutes (sampling by *scan sampling*) in the two conditions (enriched and non-enriched condition) was recorded. “No interaction” corresponds to the animals that were observed in the test area without physical contact with refuges. The behaviors were classified as inactive (absence of physical activities, such as standing, lying down or sitting without movement) or active (any behavior that involved physical activity such as locomotion or grooming). The data on the animals that were in direct contact with the refuges (exploring the surface, leaning against it or scratching it) were separated from the data on those who were simply occupying the area (Table 1).

**Table 1:** Ethogram used in Experiment I.

Interaction with Refuges	Behavior Categories	
	Active	Inactive
Physical contact with refuges (inside, over or leaning against the refuge)	Locomotion	Lying down
	Grooming	Sitting
	Surface exploring	Sleeping
	Scratching	Standing
	Rubbing	
No physical contact with refuges	Locomotion	Lying down
	Grooming	Sitting
		Sleeping
		Standing

The statistical analyses were performed using statistics software, and *p* < 0.05 was used as the threshold for significant differences. A Mann-Whitney test was used to compare the numbers of animals in the test area in the two conditions. Finally, an unpaired t test was used to compare the frequencies of active and inactive behaviors between the control and enriched conditions. The choice for a parametric test was based on the central limit theorem, which argues for a strong tendency to normality of samples with n being around 30, enabling the use of parametric tests [32].

#### 2.2.2. Experiment II: Enrichment of the Vertical Space

To examine if length of stay inside the refuges and entry frequency differed significantly between the heights of 0.0 m and 0.5 m, the Mann-Whitney test was used (the height of 1.0 m was excluded because the animals did not interact with the refuges at this height). Entry was defined as the animal putting its head and the two anterior limbs inside the refuge.

## 3. Results

### 3.1. Experiment I: Use of Horizontal Space

#### 3.1.1. Frequency of Space Utilization

**Figure 3:**
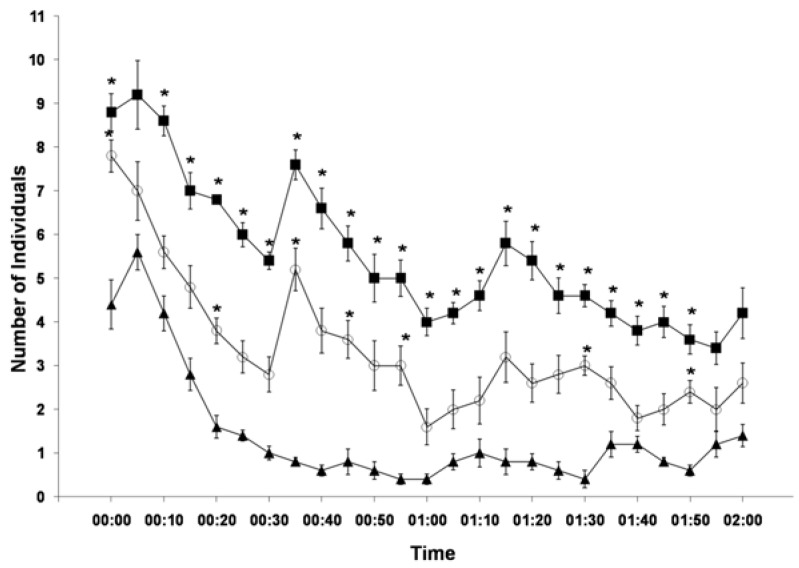
Mean number of animals observed in the test area ± standard error of the mean (*n* = 35) during the two hours of video recording in four days for the three categories of results.With enrichment (
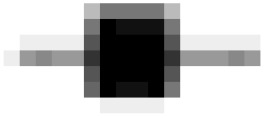
), no contact with the enrichment (
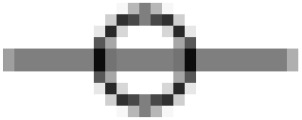
) and without enrichment (control) (
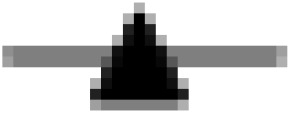
).*p < 0.05.

From the analysis of the 300 records (four days with 25 records per day for each of the three conditions), the average number of animals in the area was obtained for each sampling time for each of the three conditions: enriched, enriched without interaction and without enrichment. The values of the conditions “enriched” and “enriched without interaction” were compared with those of the condition “without enrichment”, and the highest frequencies of the use of the space were found in the enriched condition. This number differed significantly from that for the condition “without enrichment” (*p <* 0.05) in 22 of the 25 sample records (Figure 3), which corresponded to 88% of the values. In the condition “enriched without interaction”, in which only animals that were in the area but not interacting with items were recorded, significant differences from the “without enrichment” group were still found, with *p* < 0.05 for seven of the 25 sampled time points (31.82% of total samples).

#### 3.1.2. Frequencies of Active and Inactive Behaviors

**Figure 4:**
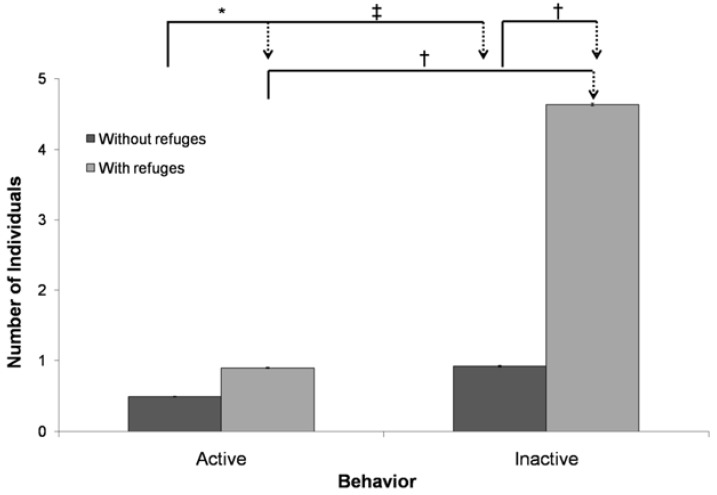
Mean numbers of individuals engaged in active and inactive behaviors in the categories “with refuges” (light bars) and “without refuges” (dark bars) during the four days of video recording in each condition for two hours. *p = 0.0019; ^†^*p* < 0.0001; ^‡^*p* = 0.0023.

In the control situation “without enrichment”, the frequency of inactive behaviors was greater than that of active behaviors (*f*_active_ = 0.49 ± 0.0065 animals/record; *f*_inactive_ = 0.92 ± 0.0104 animals/record, *p =* 0.0023). With enrichment, the frequencies of active and inactive behaviors increased significantly (*f*_active_ = 0.90 ± 0.0093 animals/record; *f*_inactive_ = 0.49 ± 0.0161 animals/record), and the inactive behaviors continued to be more frequent than the active behaviors (*p* < 0.0001), maintaining the difference between the two categories (unpaired t test, *df* = 248, *t* = 3.087, Figure 4).

### 3.2. Experiment II: Enrichment of the Vertical Space

**Figure 5:**
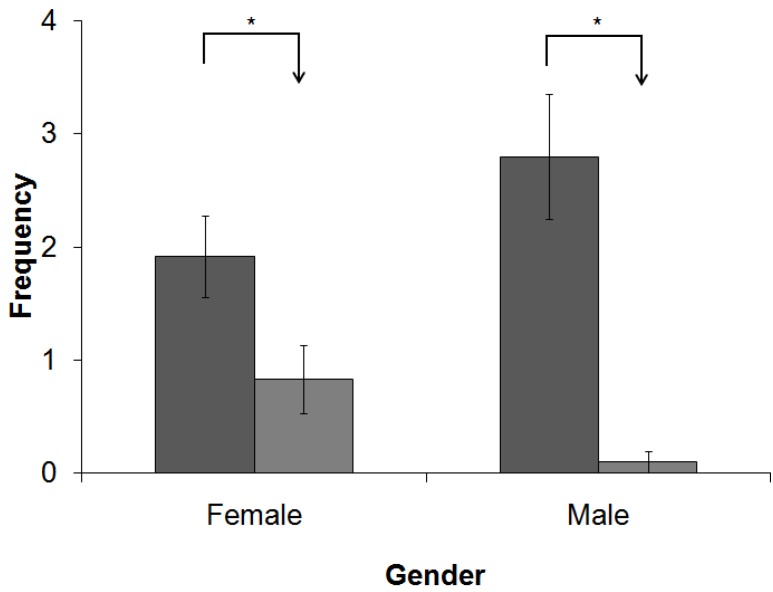
Frequency of entry into the refuges at 0.0 m (dark bars) and 0.5 m (light bars). Females and males entered the boxes at 0.0 m at significantly greater frequencies than those at 0.5 m (*p < 0.05).

**Figure 6:**
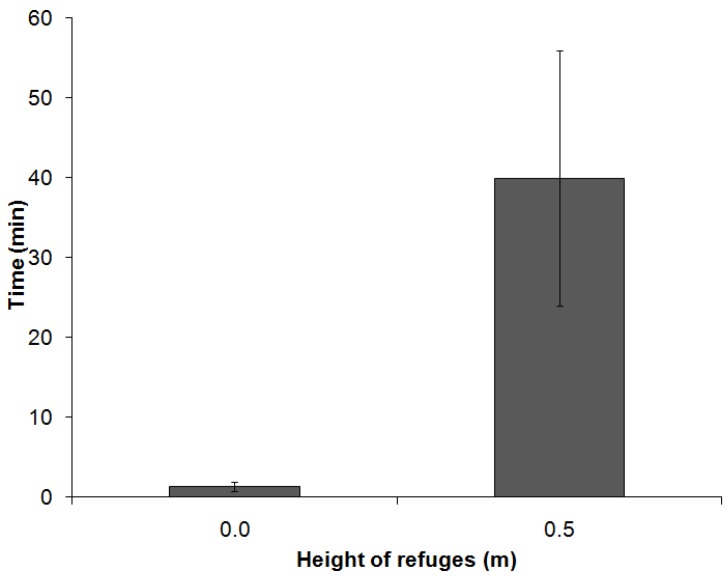
Lengths of stay of the animals within the refuges at heights of 0.0 m (*n* = 20) and 0.5 m (*n* = 7) (*p = 0.004, Mann-Whitney).

As mentioned previously, the refuges at 1.0 m height were not occupied by any animal, and therefore, the results below include only data relating to the refuges at 0.0 m and 0.5 m. The refuges set at 0.0 m were entered more frequently by males and females compared to those at 0.5 m (*p* < 0.05; the animals were separated by gender in this test to detect a possible influence of this variable on the results), as shown in Figure 5. However, the Mann-Whitney test showed that the animals spent more time occupying the refuges at 0.5 m than those at 0.0 m (*p* = 0.0041).The longest mean length of stay inside a refuge was obtained at 0.5 m (Figure 6).

**Figure 7:**
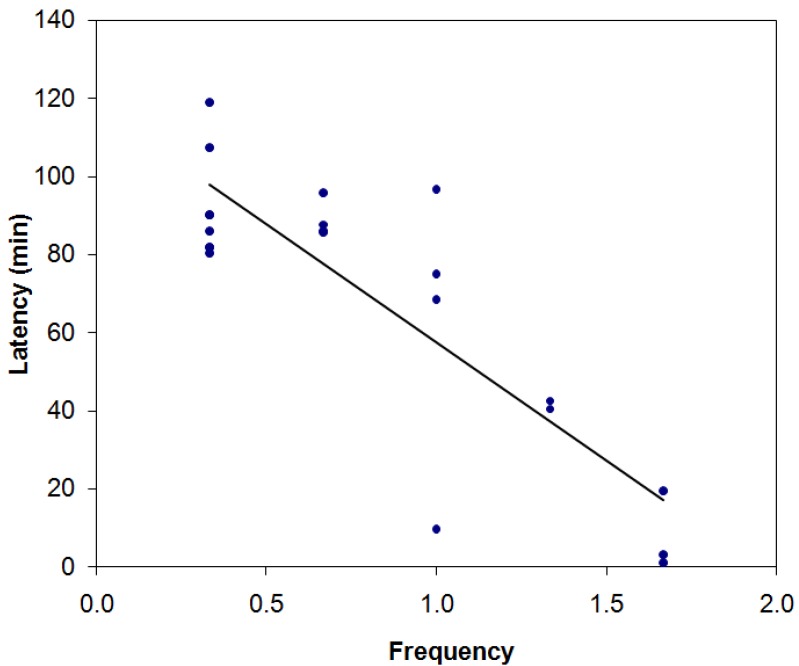
Negative correlation between the mean frequency and the latency for entry into the boxes at the two available heights (0.0 m and 0.5 m). *r* = −0.71, *p* = 0.0003. Spearman’s coefficient.

Using the Spearman coefficient to contrast the entry frequency and the length of stay in the refuges, a negative correlation (*r* = −0.71; *p* = 0.0003) was found, as shown in Figure 7. Thus, the higher the mean entry frequency into the refuges for an individual, the lower the mean value for the latency of interaction of that same individual with that enriching item.

## 4. Discussion and Conclusions

This study shows how the provision of refuges can improve the use of space previously available for a group of cats, which complements the existing literature about the benefits of this type of enrichment [25,27,30,33]. The assessment of the effects of refuges for cats in community is important given that the opportunity to hide may be responsible for reducing stress of animals kept in groups [33], especially when the physical space is restricted. Additional distinguishing features of this work is the evaluation of the use of vertically arranged refuges (frequency inputs and length of stay), the results of which are discussed in Section 4.2, and about the information about the mode of offering environmental enrichment items.

### 4.1. Experiment I: Use of Horizontal Space

The alterations in space utilization promoted by the refuges corroborate this study’s hypothesis and are in accordance with the existing literature. Laboratory rats use open areas of enclosures more frequently when they have refuges [34] and increase their exploratory behavior[35]. Likewise, vertical panels optimized the spatial distribution of poultry (*Gallus gallus domesticus*) [24]. The walls of the refuges used in this study may also have acted as vertical barriers that promoted environmental complexity,thereby contributing to animal camouflage [23]and possibly helping to create a safer environment [33,36]. This hypothesis may explain the increase in the use of the central areas of the enclosure. A large stretch of walls may increase exploratory activity [35] because the walls are capable of transforming a single environment into several different smaller spaces. In the case of domestic cats, vertical barriers seem to be important for avoiding contact with conspecifics [14].Such a reduction in the frequency of encounters between animals has also been reported after the implementation of an activity wheel, which served as a refuge in the enclosure of a marsupial species (*Sminthopsis douglasi*)[37].

Changes in the spatial distributions of animals due to the introduction of new objects can also occur because new objects increase behaviors such as hunting by stalking [36].In the case of domestic cats, such behavior is related to games, either with toys or with conspecifics. By hiding behind or even on the sides of the refuges, this behavior becomes feasible. Moreover, the opportunity to hide reduces stress in confined domestic cats [25] because they tend to hide to avoid stressful situations [28].

Active behaviors increased with the availability of refuges, proving that the refuges increased the behavioral repertoire, as occurred with broilers that had their environment enriched with perches [38]. The literature clearly shows that boxes are capable of arousing exploratory behavior in domestic cats [14]. They increase the total surface area available and fragment the environment [34],thereby increasing space between individuals.

However, an observation that had not proposed in previous studies and that was noted in the present study was that inactive behaviors increased even more than active ones. This result strengthens the hypothesis that refuges provide a safe environment for animals. The inactive behaviors reported here, including sleep and/or rest, leave the animals vulnerable to attacks by conspecifics. The refuges increased the frequency and duration of sleep in laboratory rats [34], and one of the factors contributing to the proper maintenance of cats in groups is the availability of more hidden rest places [14].

The increase in the frequency of inactive behavior also demonstrates that the enrichment with refuges did not alter the normal activity patterns of the species because they continued to exhibit patterns of inactivity during the day. Cats often react by inhibiting normal behavior when exposed to a very simple environment without enrichment [39].The fact that the animals occupied the area enriched with refuges, which had previously been underutilized, emphasizes the importance of enrichment with refuges.

The increased use of the area was significant even for animals without direct contact with the refuges, indicating an indirect effect of enrichment. Usually, the indirect effects of enrichment, such as prolonged reductions in stereotypic behaviors, are reported after its withdrawal [40].Interestingly, indirect effects occurred during the enrichment period in this study. This phenomenon is particularly important when environmental enrichment is applied to groups because the interaction of all the animals with the provided item is often not possible and may discourage its use. In rats (*Rattus norvegicus*), the level of exploratory activity may vary among individuals [41]. Enrichment methods with indirect effects such as the one used in the present study could benefit even the animals that cannot access the enrichment.

### 4.2. Experiment II: Enrichment of the Vertical Space

The results of the vertical enrichment demonstrate the importance of the method by which environmental enrichment is provided, which can influence the effectiveness of the technique by preventing or hampering its use, possibly leading to misleading conclusions about the animals’ preferences. The use of vertical space is very important for domestic cats [8,26], but when testing the use of refuges at three different heights, the highest refuge was not used the most; on the contrary, the cats did not interact with the refuges at a height of 1.0 mat all. The energy cost for the use of an item is an important factor in the execution of environmental enrichment techniques and may explain the lack of interest in the highest refuge. The animals would need to jump from the floor to access the proposed refuge, which can be done by this species. This hypothesis can be further reinforced by the fact that the animals that chose to “pay” the price of the jump to the box at 0.5 m used this refuge for longer than the cats who were content to use the refuge on the ground. This result was observed for both males and females. Thus, the use of refuges at higher levels follows an "elastic demand": as the cost increases, the demand decreases, in an analogy with human consumption [42]. However, the need may offset the cost, as in the case of chickens whose fasting time influenced their choice between an environment with food and another with a substrate that only allowed sand baths [43]. Perhaps the need to seek a higher refuge could stimulate the use of refuges at 1.0 m, in which case the cost-benefit could favor the occupation of the highest refuges in less stable social colonies. However, other studies should be performed to confirm these assumptions because there are no relevant publications in the literature.

The inverse correlation between the frequency of entry and the length of stay indicates that the animals that could immediately access the refuges generally entered them more frequently. This result reinforces the theory that individual variations exist in the ways new items are used. These variations may be caused by genetic factors [44], age or experience [42,44]. They can also result from different hierarchical levels within the colony, emphasizing the importance of offering various enrichment items to ensure that the greatest possible number of animals have access to them and thus avoiding the monopolization by a few individuals [26]. The use of environmental enrichment by only one group of animals diminishes the effectiveness of this strategy. In addition, it can potentially frustrate the animals that cannot interact with the items, further negatively affecting their welfare.

In summary, this study demonstrates the importance of environmental enrichment with refuges for welfare of cats communally housed. By expanding the use of the available space, this type of enrichment increases the likelihood of the animals performing exploratory and resting behaviors, which could compensate a limited physical space. This study also demonstrates the importance of promoting safe environments for the animals so they can perform behaviors that make them vulnerable to aggressive attacks. Simple, low cost and easily implemented enrichment strategies may be quite efficient [40], and testing them in a simple manner shows their value for each species [45]. Environmental enrichment techniques deserve careful study before implementation in a case-by-case manner because not all enrichment strategies will benefit all species [46].
